# Correction to “SAR11 clade microdiversity and activity during the early spring blooms off Kerguelen Island, Southern Ocean”

**DOI:** 10.1111/1758-2229.13151

**Published:** 2023-04-25

In this article, the axes in Figure 2 were reversed. Although the data are reported correctly in Table 1, the corrected Figure 2 is provided below:

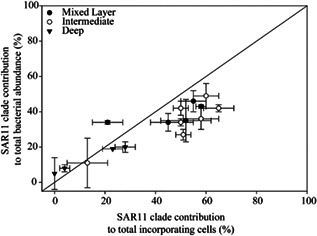

Please see the correction for the text (p909‐910): “The contribution of SAR11 to bulk leucine incorporation was **slightly higher** than expected from their contribution to abundance (Figure 2). Interestingly, the SAR11 group accounted for most of the leucine incorporating cell population, particularly in the upper layers (Table 1).”

We apologize for this error.

**REFERENCE**

Dinasquet, J., Landa, M. & Obernosterer, I. (2022) SAR11 clade microdiversity and activity during the early spring blooms off Kerguelen Island, Southern Ocean. *Environmental Microbiology Reports*, 14(6), 907–916. Available from: https://doi.org/10.1111/1758-2229.13117

